# Methyl 2-acetamido-2-(4-hy­droxy-2-methyl-1,3-dioxo-1,2,3,4-tetra­hydro­isoquinolin-4-yl)-4-methyl­penta­noate

**DOI:** 10.1107/S1600536811022999

**Published:** 2011-06-18

**Authors:** Hoong-Kun Fun, Ching Kheng Quah, Kai Xu, Yan Zhang

**Affiliations:** a X-ray Crystallography Unit, School of Physics, Universiti Sains Malaysia, 11800 USM, Penang, Malaysia; b School of Chemistry and Chemical Engineering, Nanjing University, Nanjing, 210093, People’s Republic of China

## Abstract

In the isoquinoline ring system of the title mol­ecule, C_19_H_24_N_2_O_6_, the *N*-heterocyclic ring is in a half-boat conformation. The mol­ecular structure is stabilized by an intra­molecular O—H⋯O hydrogen bond, which generates an *S*(7) ring motif. In the crystal, mol­ecules are linked *via* inter­molecular bifurcated N—H⋯(O,O) and weak C—H⋯O hydrogen bonds into a three-dimensional network.

## Related literature

For general background to and the potential biological activity of title compound, see: Yu *et al.* (2010 ▶); Huang *et al.* (2011 ▶); Rao *et al.* (1995 ▶); Nagamitsu *et al.* (1996 ▶); Evans & Weber (1986 ▶); Heimgartner (1991 ▶); Rando (1975 ▶); Griesbeck *et al.* (2003 ▶); Zhang *et al.* (2004 ▶); Wang *et al.* (2010 ▶). For the stability of the temperature controller used in the data collection, see: Cosier & Glazer (1986 ▶). For standard bond-length data, see: Allen *et al.* (1987 ▶). For ring conformations, see: Cremer & Pople (1975 ▶). For related structures, see: Fun *et al.* (2011*a* ▶,*b* ▶,*c* ▶,*d* ▶). For hydrogen-bond motifs, see: Bernstein *et al.* (1995 ▶).
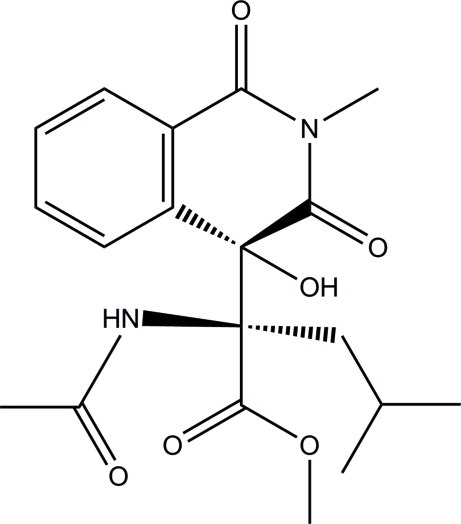


## Experimental

### 

#### Crystal data

C_19_H_24_N_2_O_6_
*M* *_r_* = 376.40
Monoclinic, 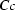

*a* = 17.8256 (15) Å
*b* = 8.7584 (6) Å
*c* = 11.9182 (8) Å
β = 100.404 (5)°
*V* = 1830.1 (2) Å^3^
*Z* = 4
Cu *K*α radiation
μ = 0.85 mm^−1^
*T* = 100 K
0.50 × 0.15 × 0.15 mm

#### Data collection

Bruker APEX DUO CCD area-detector diffractometer
Absorption correction: multi-scan (*SADABS*; Bruker, 2009 ▶) *T*_min_ = 0.678, *T*_max_ = 0.883
18268 measured reflections
2713 independent reflections
2673 reflections with *I* > 2σ(*I*)
*R*_int_ = 0.033

#### Refinement

*R*[*F* ^2^ > 2σ(*F* ^2^)] = 0.024
*wR*(*F* ^2^) = 0.059
*S* = 1.05
2713 reflections
257 parameters
2 restraints
H atoms treated by a mixture of independent and constrained refinement
Δρ_max_ = 0.11 e Å^−3^
Δρ_min_ = −0.14 e Å^−3^
Absolute structure: Flack (1983 ▶), 1204 Friedel pairs
Flack parameter: 0.05 (13)

### 

Data collection: *APEX2* (Bruker, 2009 ▶); cell refinement: *SAINT* (Bruker, 2009 ▶); data reduction: *SAINT*; program(s) used to solve structure: *SHELXTL* (Sheldrick, 2008 ▶); program(s) used to refine structure: *SHELXTL*; molecular graphics: *SHELXTL*; software used to prepare material for publication: *SHELXTL* and *PLATON* (Spek, 2009 ▶).

## Supplementary Material

Crystal structure: contains datablock(s) global, I. DOI: 10.1107/S1600536811022999/lh5262sup1.cif

Structure factors: contains datablock(s) I. DOI: 10.1107/S1600536811022999/lh5262Isup2.hkl

Supplementary material file. DOI: 10.1107/S1600536811022999/lh5262Isup3.cml

Additional supplementary materials:  crystallographic information; 3D view; checkCIF report

## Figures and Tables

**Table 1:** Hydrogen-bond geometry (Å, °)

*D*—H⋯*A*	*D*—H	H⋯*A*	*D*⋯*A*	*D*—H⋯*A*
N2—H1*N*2⋯O1^i^	0.92 (2)	2.511 (19)	3.3431 (17)	150.3 (15)
N2—H1*N*2⋯O3^i^	0.92 (2)	2.44 (2)	3.1675 (18)	135.6 (16)
O3—H1*O*3⋯O6	0.86 (2)	1.83 (2)	2.6543 (16)	160 (2)
C5—H5*A*⋯O6^ii^	0.93	2.59	3.469 (2)	157

Symmetry codes: (i) 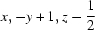; (ii) 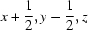.

**Fig. 1.**
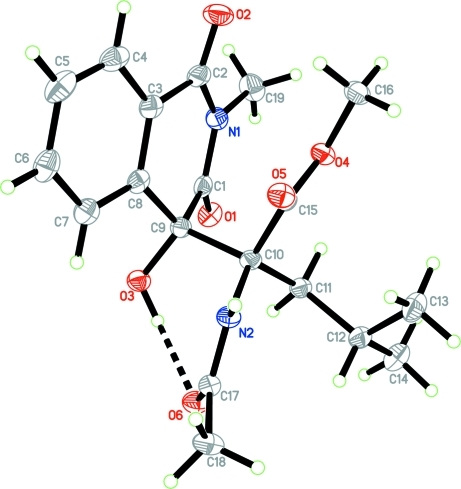
The molecular structure of the title compound showing 50% probability displacement ellipsoids for non-H atoms. Intramolecular hydrogen bond is shown as dashed line.

**Fig. 2.**
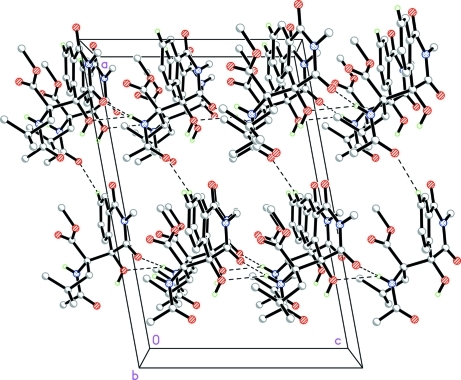
Part of the crystal structure of the title compound, viewed along the b axis. H atoms not involved in hydrogen bonds (dashed lines) have been omitted for clarity.

## supplementary crystallographic information

### Comment

Photocycloaddition of isoquinoline-1,3,4-trione combined with following transformation of the photocycloadducts has become facile method to build various scaffold containing an isoquinoline moiety (Yu *et al.*, 2010; Huang *et al.*, 2011). β-Hydroxy-amino acids are important building blocks to constitute more complex natural products. β-Hydroxy tyrosine and β-hydroxy phenylalanine derivatives are found in the clinically important antibiotic glycopeptides vancomycin, β-hydroxy leucine is found in (+)-lactacystin, and E-2-butenyl-4,N-dimethyl-l-threonine is an essential part of cyclosporine (Rao *et al.*, 1995; Nagamitsu *et al.*, 1996; Evans *et al.*, 1986). α,α-Disubstituted amino acids represent a highly interesting class of non-proteinogenic amino acids, especially in view of their potential activity as enzyme inhibitors (Heimgartner, 1991; Rando, 1975). Many bioactive natural products contain β-hydroxy-amino acid or α,α-disubstituted amino acid and there has been intense interest to develop methodology to construct such moieties using photocycloadducts of oxazoles (Griesbeck *et al.*, 2003; Zhang *et al.*, 2004; Wang *et al.*, 2010). The title compound was derived from photocycloadducts of isoquinoline-1,3,4-trione and oxazoles. Due to the importance of β-hydroxy-amino acid derivatives, we report in this paper the crystal structure of the title compound.

In the title racemic compound, Fig. 1, the isoquinoline ring system (N1/C1-C9) is not completely planar, the *N*-heterocyclic ring (N1/C1-C3/C8/C9) being distorted towards a half-boat conformation with atom C9 deviating by 0.201 (2) Å from the mean plane through the remaining atoms, puckering parameters (Cremer & Pople, 1975) Q = 0.3038 (17) Å, Θ =   70.1 (3)° and φ = 110.6 (3)°. Bond lengths (Allen *et al.*, 1987) and angles are within normal ranges and comparable to related structures (Fun *et al.*, 2011*a*,*b*,*c*,*d*). The molecular structure is stabilized by an intramolecular O3–H1O3···O6 hydrogen bond (Table 1) which generates a *S*(7) ring motif (Fig. 1, Bernstein *et al.*, 1995).

In the crystal structure, Fig. 2, molecules are linked *via* intermolecular N2–H1N2···O1^i^, N2–H1N2···O3^i^ and weak C5–H5A···O6^ii^ hydrogen bonds (Table 1) into a three-dimensional network.

### Experimental

The title compound was the main product from the acid-catalyzed transformation of the photocycloadducts of isoquinoline-1,3,4-trione and 4-isobutyl-5-methoxy-2-methyloxazole. The compound was purified by flash column chromatography with ethyl acetate/petroleum ether (1:5) as eluents. X-ray quality crystals of the title compound were obtained from slow evaporation of an acetone and petroleum ether solution (1:8) of the title compound, *m.p.* 425-427 K.

### Refinement

Atoms H1N2 and H1O3 were located in a difference Fourier map and refined freely [N2—H1N2 = 0.92 (2) Å, O3—H1O3 = 0.86 (2) Å]. The remaining H atoms were positioned geometrically and refined using a riding model with C–H = 0.93 - 0.97 Å and *U*_iso_(H) = 1.2 or 1.5 *U*_eq_(C). A rotating-group model was applied for the methyl groups. The highest residual electron density peak is located at 0.20 Å from H16B and the deepest hole is located at 0.78 Å from C9.

### Crystal data

**Table:**

C_19_H_24_N_2_O_6_	*F*(000) = 800
*M**_r_* = 376.40	*D*_x_ = 1.366 Mg m^−^^3^
Monoclinic, *C**c*	Cu *K*α radiation, λ = 1.54178 Å
Hall symbol: C -2yc	Cell parameters from 9888 reflections
*a* = 17.8256 (15) Å	θ = 5.1–64.2°
*b* = 8.7584 (6) Å	µ = 0.85 mm^−^^1^
*c* = 11.9182 (8) Å	*T* = 100 K
β = 100.404 (5)°	Needle, colourless
*V* = 1830.1 (2) Å^3^	0.50 × 0.15 × 0.15 mm
*Z* = 4	

### Data collection

**Table:**

Bruker APEX DUO CCD area-detector diffractometer	2713 independent reflections
Radiation source: fine-focus sealed tube	2673 reflections with *I* > 2σ(*I*)
graphite	*R*_int_ = 0.033
φ and ω scans	θ_max_ = 64.7°, θ_min_ = 5.1°
Absorption correction: multi-scan (*SADABS*; Bruker, 2009)	*h* = −20→20
*T*_min_ = 0.678, *T*_max_ = 0.883	*k* = −9→10
18268 measured reflections	*l* = −12→13

### Refinement

**Table:**

Refinement on *F*^2^	Secondary atom site location: difference Fourier map
Least-squares matrix: full	Hydrogen site location: inferred from neighbouring sites
*R*[*F*^2^ > 2σ(*F*^2^)] = 0.024	H atoms treated by a mixture of independent and constrained refinement
*wR*(*F*^2^) = 0.059	*w* = 1/[σ^2^(*F*_o_^2^) + (0.0319*P*)^2^ + 0.7184*P*] where *P* = (*F*_o_^2^ + 2*F*_c_^2^)/3
*S* = 1.05	(Δ/σ)_max_ < 0.001
2713 reflections	Δρ_max_ = 0.11 e Å^−^^3^
257 parameters	Δρ_min_ = −0.14 e Å^−^^3^
2 restraints	Absolute structure: Flack (1983), 1204 Friedel pairs
Primary atom site location: structure-invariant direct methods	Flack parameter: 0.05 (13)

### Special details

**Table:**

Experimental. The crystal was placed in the cold stream of an Oxford Cryosystems Cobra open-flow nitrogen cryostat (Cosier & Glazer, 1986) operating at 100.0 (1) K.
Geometry. All esds (except the esd in the dihedral angle between two l.s. planes) are estimated using the full covariance matrix. The cell esds are taken into account individually in the estimation of esds in distances, angles and torsion angles; correlations between esds in cell parameters are only used when they are defined by crystal symmetry. An approximate (isotropic) treatment of cell esds is used for estimating esds involving l.s. planes.
Refinement. Refinement of F^2^ against ALL reflections. The weighted R-factor wR and goodness of fit S are based on F^2^, conventional R-factors R are based on F, with F set to zero for negative F^2^. The threshold expression of F^2^ > 2sigma(F^2^) is used only for calculating R-factors(gt) etc. and is not relevant to the choice of reflections for refinement. R-factors based on F^2^ are statistically about twice as large as those based on F, and R- factors based on ALL data will be even larger.

### Fractional atomic coordinates and isotropic or equivalent isotropic displacement parameters (Å^2^)

**Table:**

	*x*	*y*	*z*	*U*_iso_*/*U*_eq_	
O1	0.81985 (6)	0.74615 (13)	1.04040 (10)	0.0229 (3)	
O2	1.05834 (6)	0.65801 (14)	0.97375 (10)	0.0302 (3)	
O3	0.76692 (6)	0.48621 (12)	0.94001 (10)	0.0204 (2)	
O4	0.91523 (6)	0.77211 (12)	0.76247 (9)	0.0193 (2)	
O5	0.89019 (6)	0.56633 (12)	0.65074 (9)	0.0204 (2)	
O6	0.64674 (6)	0.52211 (12)	0.77471 (10)	0.0223 (3)	
N1	0.93918 (7)	0.70763 (15)	1.00450 (11)	0.0206 (3)	
N2	0.75643 (7)	0.51865 (14)	0.70247 (12)	0.0171 (3)	
C1	0.86194 (9)	0.67556 (17)	0.99008 (14)	0.0188 (3)	
C2	0.99257 (9)	0.61559 (19)	0.96548 (14)	0.0222 (4)	
C3	0.96586 (9)	0.46387 (18)	0.91865 (14)	0.0200 (3)	
C4	1.02036 (9)	0.3555 (2)	0.90385 (14)	0.0253 (4)	
H4A	1.0719	0.3803	0.9194	0.030*	
C5	0.99735 (10)	0.2104 (2)	0.86574 (15)	0.0285 (4)	
H5A	1.0334	0.1376	0.8550	0.034*	
C6	0.92023 (10)	0.17437 (19)	0.84364 (15)	0.0259 (4)	
H6A	0.9049	0.0765	0.8192	0.031*	
C7	0.86576 (9)	0.28210 (18)	0.85750 (14)	0.0217 (3)	
H7A	0.8143	0.2565	0.8424	0.026*	
C8	0.88811 (8)	0.42821 (18)	0.89389 (13)	0.0181 (3)	
C9	0.82982 (8)	0.55120 (17)	0.90207 (14)	0.0174 (3)	
C10	0.80278 (8)	0.62935 (17)	0.77805 (14)	0.0167 (3)	
C11	0.76008 (9)	0.78137 (17)	0.78789 (13)	0.0180 (3)	
H11A	0.7260	0.7664	0.8419	0.022*	
H11B	0.7974	0.8574	0.8201	0.022*	
C12	0.71318 (9)	0.84730 (17)	0.67768 (15)	0.0217 (3)	
H12A	0.6734	0.7732	0.6477	0.026*	
C13	0.75969 (10)	0.8792 (2)	0.58470 (15)	0.0285 (4)	
H13A	0.7803	0.7852	0.5620	0.043*	
H13B	0.8006	0.9479	0.6135	0.043*	
H13C	0.7274	0.9246	0.5201	0.043*	
C14	0.67409 (10)	0.9933 (2)	0.70714 (17)	0.0306 (4)	
H14A	0.6414	1.0315	0.6401	0.046*	
H14B	0.7120	1.0686	0.7352	0.046*	
H14C	0.6443	0.9716	0.7647	0.046*	
C15	0.87344 (8)	0.65099 (17)	0.72155 (14)	0.0165 (3)	
C16	0.98383 (9)	0.79217 (19)	0.71419 (15)	0.0230 (4)	
H16A	1.0110	0.8807	0.7472	0.035*	
H16B	0.9701	0.8053	0.6331	0.035*	
H16C	1.0158	0.7037	0.7304	0.035*	
C17	0.68252 (8)	0.48575 (17)	0.69911 (14)	0.0179 (3)	
C18	0.64504 (9)	0.40140 (19)	0.59393 (15)	0.0248 (4)	
H18A	0.6056	0.3365	0.6124	0.037*	
H18B	0.6824	0.3404	0.5657	0.037*	
H18C	0.6232	0.4734	0.5365	0.037*	
C19	0.96743 (10)	0.84909 (19)	1.06333 (17)	0.0300 (4)	
H19A	0.9273	0.8958	1.0952	0.045*	
H19B	0.9839	0.9179	1.0100	0.045*	
H19C	1.0096	0.8261	1.1233	0.045*	
H1N2	0.7798 (11)	0.479 (2)	0.6459 (18)	0.025 (5)*	
H1O3	0.7237 (13)	0.510 (2)	0.8986 (18)	0.037 (6)*	

### Atomic displacement parameters (Å^2^)

**Table:**

	*U*^11^	*U*^22^	*U*^33^	*U*^12^	*U*^13^	*U*^23^
O1	0.0255 (6)	0.0248 (6)	0.0184 (7)	0.0040 (4)	0.0040 (5)	−0.0020 (5)
O2	0.0178 (6)	0.0408 (7)	0.0299 (8)	−0.0067 (5)	−0.0013 (5)	0.0021 (6)
O3	0.0173 (5)	0.0267 (6)	0.0173 (7)	−0.0020 (4)	0.0036 (5)	0.0027 (5)
O4	0.0171 (5)	0.0208 (5)	0.0201 (7)	−0.0039 (4)	0.0033 (4)	−0.0013 (5)
O5	0.0191 (5)	0.0237 (5)	0.0188 (7)	−0.0013 (4)	0.0046 (4)	−0.0021 (5)
O6	0.0177 (5)	0.0279 (6)	0.0215 (7)	−0.0011 (4)	0.0045 (5)	−0.0014 (5)
N1	0.0220 (7)	0.0223 (7)	0.0162 (8)	−0.0040 (5)	−0.0003 (5)	0.0005 (6)
N2	0.0163 (7)	0.0188 (7)	0.0161 (8)	−0.0006 (5)	0.0026 (6)	−0.0022 (5)
C1	0.0221 (8)	0.0190 (7)	0.0143 (9)	0.0005 (6)	0.0004 (6)	0.0044 (6)
C2	0.0195 (8)	0.0310 (9)	0.0143 (9)	−0.0008 (6)	−0.0019 (6)	0.0057 (7)
C3	0.0206 (8)	0.0281 (8)	0.0112 (9)	0.0010 (6)	0.0024 (6)	0.0038 (7)
C4	0.0210 (8)	0.0383 (10)	0.0163 (10)	0.0049 (7)	0.0027 (7)	0.0045 (7)
C5	0.0322 (10)	0.0339 (10)	0.0193 (11)	0.0139 (7)	0.0042 (7)	0.0002 (7)
C6	0.0352 (10)	0.0242 (9)	0.0168 (10)	0.0055 (7)	0.0009 (7)	0.0007 (7)
C7	0.0248 (8)	0.0246 (8)	0.0151 (9)	0.0009 (7)	0.0021 (6)	−0.0002 (7)
C8	0.0210 (8)	0.0239 (8)	0.0095 (9)	0.0021 (6)	0.0030 (6)	0.0028 (6)
C9	0.0167 (7)	0.0204 (8)	0.0149 (9)	−0.0010 (6)	0.0025 (6)	0.0017 (6)
C10	0.0161 (7)	0.0196 (7)	0.0130 (9)	−0.0030 (6)	−0.0007 (6)	−0.0009 (6)
C11	0.0159 (7)	0.0216 (8)	0.0163 (9)	0.0001 (6)	0.0025 (6)	0.0005 (6)
C12	0.0204 (7)	0.0219 (8)	0.0207 (10)	−0.0012 (6)	−0.0015 (6)	0.0013 (7)
C13	0.0318 (9)	0.0316 (9)	0.0212 (10)	0.0051 (7)	0.0022 (7)	0.0070 (8)
C14	0.0318 (9)	0.0302 (9)	0.0280 (12)	0.0085 (7)	0.0002 (8)	0.0036 (8)
C15	0.0156 (7)	0.0191 (8)	0.0134 (9)	−0.0003 (6)	−0.0010 (6)	0.0030 (6)
C16	0.0190 (8)	0.0264 (8)	0.0238 (10)	−0.0048 (6)	0.0043 (7)	0.0010 (7)
C17	0.0165 (7)	0.0178 (7)	0.0191 (10)	−0.0003 (6)	0.0018 (6)	0.0013 (6)
C18	0.0178 (8)	0.0319 (9)	0.0241 (10)	−0.0055 (7)	0.0024 (7)	−0.0034 (8)
C19	0.0364 (10)	0.0282 (9)	0.0235 (10)	−0.0103 (7)	0.0004 (8)	−0.0038 (8)

### Geometric parameters (Å, °)

**Table:**

O1—C1	1.211 (2)	C8—C9	1.512 (2)
O2—C2	1.217 (2)	C9—C10	1.621 (2)
O3—C9	1.4032 (19)	C10—C15	1.543 (2)
O3—H1O3	0.86 (2)	C10—C11	1.549 (2)
O4—C15	1.3368 (18)	C11—C12	1.537 (2)
O4—C16	1.4531 (18)	C11—H11A	0.9700
O5—C15	1.201 (2)	C11—H11B	0.9700
O6—C17	1.236 (2)	C12—C13	1.525 (3)
N1—C1	1.3851 (19)	C12—C14	1.527 (2)
N1—C2	1.390 (2)	C12—H12A	0.9800
N1—C19	1.467 (2)	C13—H13A	0.9600
N2—C17	1.3420 (19)	C13—H13B	0.9600
N2—C10	1.4715 (19)	C13—H13C	0.9600
N2—H1N2	0.92 (2)	C14—H14A	0.9600
C1—C9	1.548 (2)	C14—H14B	0.9600
C2—C3	1.486 (2)	C14—H14C	0.9600
C3—C4	1.392 (2)	C16—H16A	0.9600
C3—C8	1.399 (2)	C16—H16B	0.9600
C4—C5	1.386 (3)	C16—H16C	0.9600
C4—H4A	0.9300	C17—C18	1.504 (2)
C5—C6	1.389 (3)	C18—H18A	0.9600
C5—H5A	0.9300	C18—H18B	0.9600
C6—C7	1.385 (2)	C18—H18C	0.9600
C6—H6A	0.9300	C19—H19A	0.9600
C7—C8	1.386 (2)	C19—H19B	0.9600
C7—H7A	0.9300	C19—H19C	0.9600
			
C9—O3—H1O3	113.6 (14)	C10—C11—H11A	108.0
C15—O4—C16	113.89 (13)	C12—C11—H11B	108.0
C1—N1—C2	124.49 (13)	C10—C11—H11B	108.0
C1—N1—C19	118.57 (13)	H11A—C11—H11B	107.2
C2—N1—C19	116.93 (13)	C13—C12—C14	110.13 (14)
C17—N2—C10	126.82 (13)	C13—C12—C11	114.00 (13)
C17—N2—H1N2	117.8 (12)	C14—C12—C11	108.57 (14)
C10—N2—H1N2	114.8 (12)	C13—C12—H12A	108.0
O1—C1—N1	121.72 (14)	C14—C12—H12A	108.0
O1—C1—C9	120.54 (14)	C11—C12—H12A	108.0
N1—C1—C9	117.67 (13)	C12—C13—H13A	109.5
O2—C2—N1	120.25 (15)	C12—C13—H13B	109.5
O2—C2—C3	122.84 (15)	H13A—C13—H13B	109.5
N1—C2—C3	116.87 (13)	C12—C13—H13C	109.5
C4—C3—C8	120.56 (15)	H13A—C13—H13C	109.5
C4—C3—C2	118.28 (14)	H13B—C13—H13C	109.5
C8—C3—C2	121.12 (14)	C12—C14—H14A	109.5
C5—C4—C3	119.65 (16)	C12—C14—H14B	109.5
C5—C4—H4A	120.2	H14A—C14—H14B	109.5
C3—C4—H4A	120.2	C12—C14—H14C	109.5
C4—C5—C6	119.64 (16)	H14A—C14—H14C	109.5
C4—C5—H5A	120.2	H14B—C14—H14C	109.5
C6—C5—H5A	120.2	O5—C15—O4	123.60 (14)
C7—C6—C5	120.92 (16)	O5—C15—C10	123.84 (13)
C7—C6—H6A	119.5	O4—C15—C10	112.54 (13)
C5—C6—H6A	119.5	O4—C16—H16A	109.5
C6—C7—C8	119.87 (15)	O4—C16—H16B	109.5
C6—C7—H7A	120.1	H16A—C16—H16B	109.5
C8—C7—H7A	120.1	O4—C16—H16C	109.5
C7—C8—C3	119.33 (14)	H16A—C16—H16C	109.5
C7—C8—C9	121.04 (13)	H16B—C16—H16C	109.5
C3—C8—C9	119.57 (14)	O6—C17—N2	123.78 (14)
O3—C9—C8	109.28 (12)	O6—C17—C18	121.61 (13)
O3—C9—C1	106.64 (13)	N2—C17—C18	114.60 (14)
C8—C9—C1	111.74 (12)	C17—C18—H18A	109.5
O3—C9—C10	109.99 (12)	C17—C18—H18B	109.5
C8—C9—C10	109.67 (13)	H18A—C18—H18B	109.5
C1—C9—C10	109.47 (12)	C17—C18—H18C	109.5
N2—C10—C15	103.07 (12)	H18A—C18—H18C	109.5
N2—C10—C11	112.55 (12)	H18B—C18—H18C	109.5
C15—C10—C11	112.23 (12)	N1—C19—H19A	109.5
N2—C10—C9	108.76 (12)	N1—C19—H19B	109.5
C15—C10—C9	108.54 (11)	H19A—C19—H19B	109.5
C11—C10—C9	111.30 (13)	N1—C19—H19C	109.5
C12—C11—C10	117.22 (13)	H19A—C19—H19C	109.5
C12—C11—H11A	108.0	H19B—C19—H19C	109.5
			
C2—N1—C1—O1	168.38 (15)	O1—C1—C9—C8	−151.28 (14)
C19—N1—C1—O1	−12.6 (2)	N1—C1—C9—C8	31.84 (19)
C2—N1—C1—C9	−14.8 (2)	O1—C1—C9—C10	87.02 (17)
C19—N1—C1—C9	164.27 (14)	N1—C1—C9—C10	−89.86 (15)
C1—N1—C2—O2	174.17 (15)	C17—N2—C10—C15	−164.63 (14)
C19—N1—C2—O2	−4.9 (2)	C17—N2—C10—C11	−43.5 (2)
C1—N1—C2—C3	−8.2 (2)	C17—N2—C10—C9	80.31 (18)
C19—N1—C2—C3	172.78 (14)	O3—C9—C10—N2	−49.92 (16)
O2—C2—C3—C4	12.4 (2)	C8—C9—C10—N2	70.29 (15)
N1—C2—C3—C4	−165.20 (15)	C1—C9—C10—N2	−166.77 (12)
O2—C2—C3—C8	−169.92 (15)	O3—C9—C10—C15	−161.37 (12)
N1—C2—C3—C8	12.5 (2)	C8—C9—C10—C15	−41.16 (16)
C8—C3—C4—C5	−0.9 (2)	C1—C9—C10—C15	81.77 (14)
C2—C3—C4—C5	176.79 (16)	O3—C9—C10—C11	74.64 (15)
C3—C4—C5—C6	−0.5 (3)	C8—C9—C10—C11	−165.15 (12)
C4—C5—C6—C7	1.0 (3)	C1—C9—C10—C11	−42.22 (16)
C5—C6—C7—C8	0.0 (3)	N2—C10—C11—C12	−42.05 (18)
C6—C7—C8—C3	−1.4 (2)	C15—C10—C11—C12	73.68 (17)
C6—C7—C8—C9	175.79 (15)	C9—C10—C11—C12	−164.44 (12)
C4—C3—C8—C7	1.9 (2)	C10—C11—C12—C13	−58.65 (18)
C2—C3—C8—C7	−175.74 (15)	C10—C11—C12—C14	178.18 (13)
C4—C3—C8—C9	−175.38 (15)	C16—O4—C15—O5	−0.5 (2)
C2—C3—C8—C9	7.0 (2)	C16—O4—C15—C10	177.71 (12)
C7—C8—C9—O3	37.1 (2)	N2—C10—C15—O5	−15.55 (19)
C3—C8—C9—O3	−145.72 (14)	C11—C10—C15—O5	−136.90 (15)
C7—C8—C9—C1	154.86 (15)	C9—C10—C15—O5	99.66 (17)
C3—C8—C9—C1	−27.9 (2)	N2—C10—C15—O4	166.23 (12)
C7—C8—C9—C10	−83.55 (17)	C11—C10—C15—O4	44.89 (17)
C3—C8—C9—C10	93.64 (17)	C9—C10—C15—O4	−78.55 (14)
O1—C1—C9—O3	−31.9 (2)	C10—N2—C17—O6	−15.0 (2)
N1—C1—C9—O3	151.20 (13)	C10—N2—C17—C18	164.21 (14)

### Hydrogen-bond geometry (Å, °)

**Table:**

*D*—H···*A*	*D*—H	H···*A*	*D*···*A*	*D*—H···*A*
N2—H1N2···O1^i^	0.92 (2)	2.511 (19)	3.3431 (17)	150.3 (15)
N2—H1N2···O3^i^	0.92 (2)	2.44 (2)	3.1675 (18)	135.6 (16)
O3—H1O3···O6	0.86 (2)	1.83 (2)	2.6543 (16)	160 (2)
C5—H5A···O6^ii^	0.93	2.59	3.469 (2)	157

Symmetry codes: (i) *x*, −*y*+1, *z*−1/2; (ii) *x*+1/2, *y*−1/2, *z*.
